# Using Environmental Simulations to Test the Release of Hazardous Substances from Polymer-Based Products: Are Realism and Pragmatism Mutually Exclusive Objectives?

**DOI:** 10.3390/ma13122709

**Published:** 2020-06-15

**Authors:** Nicole Bandow, Michael D. Aitken, Anja Geburtig, Ute Kalbe, Christian Piechotta, Ute Schoknecht, Franz-Georg Simon, Ina Stephan

**Affiliations:** 1 German Environment Agency, Corrensplatz 1, 14195 Berlin, Germany; nicole.bandow@uba.de; 2 Department of Environmental Sciences and Engineering, University of North Carolina, Chapel Hill, NC 27599-7431, USA; mike_aitken@unc.edu; 3 Bundesanstalt für Materialforschung und-prüfung (BAM), 12200 Berlin, Germany; anja.geburtig@bam.de (A.G.); Christian.piechotta@bam.de (C.P.); ute.schoknecht@bam.de (U.S.); franz-georg.simon@bam.de (F.-G.S.); ina.stephan@bam.de (I.S.)

**Keywords:** environmental simulations, polymer-based products, artificial weathering, degradation, leaching, soil contact

## Abstract

The potential release of hazardous substances from polymer-based products is currently in the focus of environmental policy. Environmental simulations are applied to expose such products to selected aging conditions and to investigate release processes. Commonly applied aging exposure types such as solar and UV radiation in combination with water contact, corrosive gases, and soil contact as well as expected general effects on polymers and additional ingredients of polymer-based products are described. The release of substances is based on mass-transfer processes to the material surfaces. Experimental approaches to investigate transport processes that are caused by water contact are presented. For tailoring the tests, relevant aging exposure types and release quantification methods must be combined appropriately. Several studies on the release of hazardous substances such as metals, polyaromatic hydrocarbons, flame retardants, antioxidants, and carbon nanotubes from polymers are summarized exemplarily. Differences between natural and artificial exposure tests are discussed and demonstrated for the release of flame retardants from several polymers and for biocides from paints. Requirements and limitations to apply results from short-term artificial environmental exposure tests to predict long-term environmental behavior of polymers are presented.

## 1. Introduction

Many materials are exposed to the ambient environment, so that there is a need to understand how such exposure might affect the environment and vice versa. To come up to growing demands, plastics must be optimized using several additives. As long as such additives stay within the materials over the whole life cycle, they do not pose a problem to the environment, even if they are classified as hazardous. Whether, and if so in what quantity, such substances are released into the environment during a product’s service life, depends on the conditions of use. For example, building materials can be exposed to the ambient atmosphere, sunlight, and precipitation, can be in direct contact with soil, or can be submerged in groundwater or marine environments. Government regulatory agencies can be concerned with the release of potentially harmful substances embedded in or on the surface of a material, such as a biocide in paint. Manufacturers, on the other hand, might be interested in the reciprocal impact of environmental exposure on the material itself, which could influence its longevity and/or efficacy. The interface of these two perspectives—how releasing a key component to the environment affects the material and how changes to material properties due to environmental exposure influence the release of a chemical of concern—can also be important. For purposes of this review, we use the term “chemical” to mean a substance that either was added to a material by the manufacturer, is a residual constituent of the manufacturing process, or is a product resulting from transformation of the material upon environmental exposure. From an environmental perspective, such chemicals might be regarded as contaminants, whereas to the manufacturer they may be key ingredients necessary to achieve the desired material properties, unanticipated byproducts of material manufacture, or unavoidable transformation products resulting from normal service in the field.

Regulatory agencies often require testing to evaluate the potential environmental impact of a material exposed to the ambient environment. Standardized tests have been developed for such purposes, either by the agency itself or by standardization bodies (such as the International Organization for Standardization, ISO). In some cases, there might not be a standardized test, but protocols have been developed that are widely adopted. Apart from regulatory constraints, manufacturers concerned about effects of the environment on a particular material or an ingredient in a material can have more freedom to develop de novo tests, though more in the context of research and development than in creating a standard protocol.

The development of a standardized test is typically subject to considerable stakeholder involvement, with the aim to optimize relevance while accounting for user concerns regarding time and cost. Although environmental exposures occur over a period of years, testing is by necessity conducted over much shorter time scales. Short-term tests can also be useful to quickly compare the potential impacts on two or more materials.

A typical question, then, in developing a standard testing method is how to condense the time frame for a certain type of environmental exposure (such as exposure to ambient precipitation) into a time frame that is both practical and cost-effective. Doing this inherently involves tradeoffs between expediency and the extent to which the resulting test fits the purpose for which it was designed. The results of such tests are the basis for regulatory processes. How to assess the results, e.g., development of criteria for assessment, will not be considered here.

Plastics are currently in the focus of environmental policy due to their long-term behavior and therefore to their persistence. Not only do they appear as visible particles in the sea and on the beach, the almost unknown behavior of their additives and the related transformation products are of environmental concern. Therefore, this review focuses on the simulation of the environmental behavior of polymer-based products. Examples of such studies carried out by the authors are given. To our knowledge the combination of environmental exposure of polymer-based products to induce aging processes with the transfer of chemicals to environmental compartments in one experiment is quite rare. We believe that this combination is crucial for a better understanding and risk assessment and is therefore highlighted in this review article.

**Figure 1:**
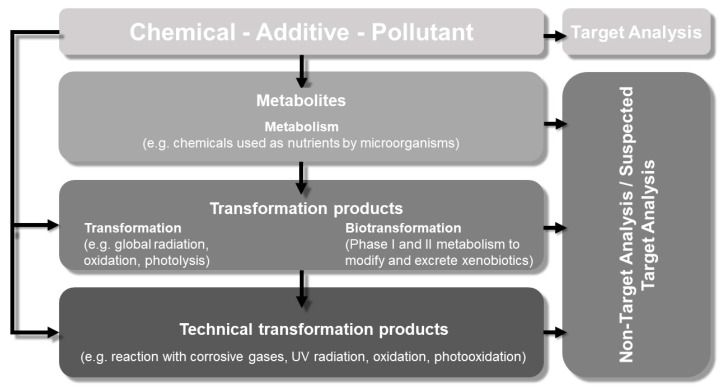
Possible consequence for chemicals under environmental relevant conditions in general.

Photochemical, chemical, and biological processes have the potential to transform a chemical in a material into one or more products whose identities and properties might not be known yet, but might be of environmental concern [1,2,3] (Figure 1). Such products are often overlooked because material-testing methods are usually designed to test known (target) substances. In some cases, bioassays [4] intended to detect the potential effect(s) of such products might be required by a regulatory agency [5]. Important are not only the chemicals that polymers originally consist of, but also the compounds that are processed via structural transformation in the environment or by organisms [6]. Abiotic processes (e.g., photolysis, hydrolysis, oxidation) can modify or degrade substances to structurally altered species. Biodegradation and biodeterioration are the interactions of microorganisms or other organisms with materials [7]. The biodegradation of organic matter is the result of the action of a multitude of different organisms. In all stages, microorganisms and their metabolism play an important role as they both use organic matter as a source of nutrients and their enzymes can result in other chemical changes not linked directly to their use of a substrate. They can also cause degradation of a material by colonizing its surface and causing physicochemical changes (e.g., in pH, moisture conditions) that leads to abiotic degradation. The huge diversity within biological systems allows them to contribute to the degradation of a wide range of materials, including many synthetic polymers.

The degradation of polymers is a known reason for the release of additives and other chemicals from the material into the environment [5,8]. For the quantification of released chemicals that are known, for example, additives such as stabilizers, a target-analytical approach must be established. For the identification, characterization, and subsequent quantification of possible transformation products or metabolites, a non-target- or suspected-target-analysis strategy is essential [9].

## 2. Aging Exposure Types

Often, the release of hazardous substances changes with the aging status of the plastic material. Aging—which is defined as the entirety of irreversible chemical and physical processes within a material over time [10]—can be caused by several natural environmental conditions. This chapter summarizes the nature-oriented simulations of such processes.

### 2.1. Weathering

For polymeric systems, weathering exposure causes mainly photochemical aging [11,12,13]. Solar UV radiation can split organic bonds. In the presence of oxygen, this typically leads to photo-oxidation, including e.g., chain scissions. The kinetics of the photochemical processes greatly depends on the temperature. Besides UV irradiance and temperature, also rain and humidity can have a major influence on the weathering results. Both can lead to hydrolytic degradation of some polymeric materials. Moreover, cyclic moisture conditions can generate mechanical stresses and accelerate migration processes.

There are several concepts for simulating outdoor weathering effects in artificial laboratory tests; each has specific advantages and disadvantages.

In Xenon arc devices [14]—with a spectral irradiance distribution closely resembling that of solar radiation—the high noon scenario is mapped, due to radiation heating mainly from the IR and VIS radiation amounts [15]. On the one hand, this reflects the maximum differences in surface temperatures between different colors [16]; on the other hand, it neglects the moisture parameter, which can be important for e.g., migration, crack formation or abrasion due to mechanical stresses, or PVC discoloration. Here—only in the presence of moisture—the temporarily generated polyenes are oxidized by the photocatalytic action of TiO_2_ under wet conditions; under the too-dry Xenon arc conditions, these polyenes remain, noticeable as an unrealistic, strong discoloration [17]. In Xenon arc devices, the temperature gradient over the thickness occurs similarly to high noon natural weathering conditions, but it varies with many parameters. These include not only IR radiation and IR absorption, but also the material’s thickness and thermal conductivity, as well as fluid dynamic processes, which greatly depend on the installation position and environment.

In various UV fluorescent lamp devices [18], a temperature gradient is induced by rear-contacting to the cooler laboratory temperature, in order to generate dew during the dark wetting times—a concept that works quite well for metal coating panels, but not for thicker plastic specimens. During the irradiation, the specimen’s surface is heated by convection. Unfortunately, this can lead to huge temperature variations, depending on the kind of temperature control and the sample thickness [19].

However, due to the negligible radiation heating, the use of UV fluorescent lamps enables a very accurate temperature control over the whole specimen (surface as well as bulk) by integrating the UV lamps into a climate chamber, excluding the complex fluid dynamic effects. On the one hand, this concept enables accurate control of the surface climate. On the other hand, a large temperature and humidity range can be adjusted at the sample surface [20].

Due to the strong and individually different interactions, standardized artificial weathering tests are quite pragmatic. Although generally aiming at acceleration, no acceleration factor compared with any outdoor exposure can be generalized for different polymers. There are various objectives in the conceptual design of such artificial weathering tests: e.g., the determination of acceleration factors [21], the comparison of the ranking of different materials in regard to aging effects [22,23], or the identification of potential failure locations for components.

### 2.2. UV Radiation in Terms of Photocatalysis or Photocatalytic Effects

The degradation of emerging pollutants in the environment especially in the context of so-called AOP (advanced oxidation processes) is of great interest and was investigated in many scientific studies. Consequently, chemicals or pollutants released from aged materials can be degraded into distinct transformation products.

For photocatalytic reactions in terms of AOP titanium oxide is the most common compound. Here, titanium (IV) dioxide is the species of interest. It is chemically inert and can only be dissolved in sulfuric of hydrofluoric acid. When illuminated with UV radiation, photocatalytic radical reactions can take place by generating free charge carriers, electrons in the conduction band and holes in the valence band. Normally, these charge carrier pairs recombine very quickly; however, due to the band bending in the area of the surface, charge carriers can be separated. These generally react with adsorbed oxygen and water to form hydroxyl and peroxy radicals to degrade or damage polymer-based materials and their additives [24].

Polypropylene (PP), for example, is used in food packaging. Due to its inertness, it is commonly not biodegradable and resists microbial or enzymatic degradation. Titanium dioxide can be used to accelerate the photodegradation of synthetic polymers, resulting in the release of other components or additives in the polymer with subsequent transformation processes, releasing a various number of transformation products into the environment [25,26].

### 2.3. Ozone as an Example of a Corrosive Gas

Ozone cracking, or the interaction of ozone with materials such as polymers, will attack double bonds in rubber chains or other polymers [27]. Natural rubber and polyunsaturated polymers like, polybutadiene can be corroded and finally degraded. Here, the concentration of ozone acting as a corrosive gas is in direct correlation to the extend or degree of degradation. This kind of degradation or decomposition of polymers enables the release of imbedded chemicals such as stabilizers, additives, or other pollutants to the environment. This test can be performed according to the standard ASTM D1149-18 [28].

### 2.4. Soil Bed

Soil bed tests are used when the materials in their use phase will either be directly exposed to the soil or heavy organic soiling has to be expected (e.g., outdoor textiles [29,30], buffers of cars). Any soil bed is basically a black box concerning the soil organisms (e.g., bacteria, fungi, algae, mites, nematodes). Depending on the initial pH of the soil and material, the degree of moisture, and the temperature, these organisms are more or are less active. While some might be in a dormant stage (e.g., as spores), others might find their ecological niche under precisely these circumstances.

The greater the biological diversity in a soil bed, the more likely it is that a material inserted in such an environment will be colonized or even deteriorated. Qualifying and quantifying the different groups of organisms in a soil bed is an immensely time-consuming and expensive undertaking, and the results are short-lived because adding a new material can change the community at the interface of material and soil. Communities also fluctuate when events such as rain or dryness leave pores in the soil structure, whether aerobic or anaerobic. On a locally very small scale, changes in pH can occur, as fungi in particular emit organic acids as secondary metabolites.

To gain some control over such a complex test setup, it is common practice to include either degradable materials (e.g., cotton textile, degradable wood species) and to monitor their deterioration or to measure sum parameters such as O_2_ consumption or CO_2_ output during an experiment to document metabolism. When using degradable materials, these serve as a reference benchmark to tell of a certain capacity the soil showed over a longer period, as most soil bed tests are performed for from at least 4 weeks up to several years. Withdrawing reference materials after time intervals makes it possible to monitor some aspects of the capacity of a soil bed. Mainly, strength loss, mass loss, or changes of the appearance of the surface are measured to rate or to quantify the impact of a soil bed on a material.

Soil containing a high degree of variability of organisms is required for this test, and the biological activity must be maintained during the test. To achieve an acceleration of effects between material and soil, two basic principles are important: (i) keep the moisture level in the soil so that neither dryness nor waterlogging occurs, (ii) do not allow temperatures below 15 °C or above 35 °C. Fine-tuning temperature and wetness makes it possible to tailor the soil bed to different use conditions. If comparable results are to be obtained, it is necessary to use soil of the same physical and chemical description regarding e.g., particles size, water holding capacity, and pH.

## 3. Mass-Transfer Processes

Material-testing protocols are generally designed to evaluate the transfer of a chemical from the material to the environmental compartment of interest (air, water, soil). A leaching procedure, for example, might be developed to reasonably approximate the total volume of water to which the material might be exposed over a defined period of interest, but condensed into a short-term test designed to reproduce the total volume per unit surface area or sample mass.

To analyze the release, two different approaches are possible: (i) as a direct proof, the amount of the released substance during the exposure can be measured, or (ii) as an indirect proof, the remaining concentration after several exposures is compared to the substance’s original amount within the specimen. Such residual analysis is not always possible, e.g., the release of bisphenol A only develops during polycarbonate aging [31,32,33]. Either the water samples can be directly analyzed or the analytes can be enriched by passive sampling techniques in the experiments [34] or afterwards by techniques such as solid phase extraction or stir bar sorptive extraction [35].

To investigate the release during the exposure of polymer sheets in a weathering chamber, the accumulated concentration within the circulated rainwater can be measured, using a circulation system to spray water. For the investigation of polar substances, the circulated water can be sampled and analyzed after various periods. A main disadvantage of this method is that sorption of the substance somewhere within the device, such as at seals or pipes, is hardly preventable. To avoid this, as many stainless steel sections as possible should be used, e.g., the water tank. Another possibility is the separation of aging and leaching into two consecutive steps. To investigate nonpolar species, silicone-based passive samplers can be placed in the water drainage to enrich the analytes [36].

Transformation processes of analytes in the material itself or after release in the eluates further complicates the analysis. The method of sample preparation or analysis may be not suitable for transformation products and can be adapted only after the identification of chemicals. This identification procedure is time-consuming and usually does not identify all unknown compounds in a sample [37]. If both the residual content within the specimen and the release during the exposure can be measured with sufficient precision, a mass balance can be established; here, all other possible losses, such as sorption [38] or degradation [39], should be considered.

Various leaching tests, such as tank tests, batch tests, and column percolation tests, have been developed to measure the mass transfer by contact with water. The choice of test depends on the material properties, as well as on the intended application of the material under investigation. Monolithic and sheet-like products are usually tested in tank tests (e.g., [40,41,42]), while batch tests (agitation by shaking in a bottle, e.g., [43,44]) and up-flow column percolation tests have been successfully established (e.g., [45,46]) for granular materials. However, some tests, in particular batch tests and one-stage tank tests developed for compliance testing, are less suitable for the investigation of long-term mass-transfer processes because the conditions in the test are not comparable to field scenarios (e.g., contact time between sample and leachant, liquid-to-solid ratio). For granular materials, column percolation tests are an appropriate tool for investigating the long-term contaminant transfer as e.g., organic compounds [47] from a variety of materials as reconstruction products [48], waste [49] and fly ash and slag [50] to soil and water.

Tank tests may include permanent immersion (e.g., dynamic surface leaching test, DSLT, CEN/TS 16637-2 [40]) or intermittent immersion at defined ratios between the exposed surface area and the volume of water used for leaching (EN 16105 [41]). Eluate concentrations can be transferred to emissions per surface area if needed to assess results. The test design of the DSLT includes increasing leaching intervals and enables the identification of diffusion-controlled leaching processes. Intermittent immersion tests with a sequence of dry and wet cycles are closer to realistic exposure conditions and reproduce mass-transfer processes in the material also during dry periods [51].

**Figure 2:**
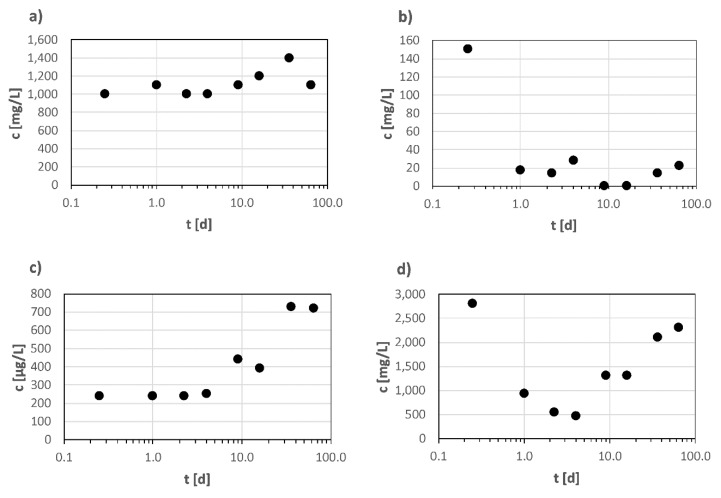
Examples of concentration patterns in elute fractions from the dynamic surface leaching test that indicate different release mechanisms as described in [40]. On the *x*-axis, the period of water exchange in days is shown for (**a**) a process controlled by water solubility, (**b**) first flush (**c**) a diffusion-controlled process, and (**d**) first flush combined with a diffusion-controlled process.

Mass transfer to water includes various mechanisms that determine the concentration’s time profile. Often not only a single mechanism but rather a shift from one dominating mechanism to another during longer leaching experiments is observed [52,53]. As an example, in a CEN TC 351 standard [40], different mechanisms are described: for compounds with low water solubility, maximum release is limited, which is often indicated by almost constant eluate concentrations (Figure 2a). At the beginning, a first flush or surface wash-off is observed if compounds with high water solubility are loosely attached to material surfaces (Figure 2b). High eluate concentrations for the first fraction are accompanied by rapidly decreasing concentrations, especially in cases of a rapid depletion due to limited stock in the material. Depletion proceeds from the surface to the inner layers, establishing a concentration gradient responsible for diffusion processes in the material itself. Diffusion-controlled processes are proportional to the square root of contact time and are indicated by the pattern of the eluate concentrations after the defined periods of water contact (Figure 2c). Figure 2d shows a combination of first flush and diffusion. Diffusion in bulk material is often slow. Inhomogeneous materials hamper a mechanistic description of diffusion processes, as only overall diffusion rates for the bulk material are determined [54]. Pores filled with water enhance diffusion within the material, but important for mass transfer is not only the total porosity, but also the structure of the pore network e.g., dead ends and surface area [55], linkage between the pores [56]. A higher influence on such processes by the shape of pores than their sizes was found by Al-Raoush [57]. Molecular size, existing water-filled pores, and water solubility are substance parameters influencing the diffusion rate.

The velocity of mass transfer at the solid/water interface is driven mainly by the concentration gradient between the two phases and will slow when equilibrium is reached [58,59,60]. The thickness of the water boundary layer between the two phases depends on the turbulence in the system. In situations where the liquid phase is not agitated, diffusion through this layer may become the rate-limiting step [59]. For column percolation tests, equilibrium is rapidly established at the beginning of a test for many materials in a broad range of particle size distributions and flow velocities [61,62], but mostly cannot be maintained throughout the test [63]. Achievement of equilibrium is important if the results from laboratory leaching tests are to be comparable to field studies [61,64].

## 4. Tailoring Exposure Tests—Examples

To accelerate the release of a substance in an artificial environmental test, some knowledge is needed of the release mechanism, the degradation behavior of the material, and the limits of the relevant environmental factors. Based on this knowledge, several exposure types can be combined in one exposure test, e.g., in weathering chambers.

For the release of additives or aging products during weathering exposure of polymeric systems [65], two effects must be considered. On the one hand, the polymeric matrix degrades due to chemical aging, probably uncovering embedded ingredients. On the other hand, cyclic stress can greatly increase the migration effects of various components.

To accelerate the release of additives or aging products, an artificial weathering test should respect both effects, but still keep the exposure parameters within natural limits.

To increase mechanical stresses, long wetting periods can be included, which increase the moisture penetration depth. Additionally, thermal stress can be applied in artificial weathering tests by varying the temperature levels. To accelerate polymer matrix degradation, high temperatures should be chosen. Doing so, both maximum outdoor temperatures and possible transition temperatures of the polymeric system should be considered, to avoid unrealistic natural effects.

**Figure 3:**
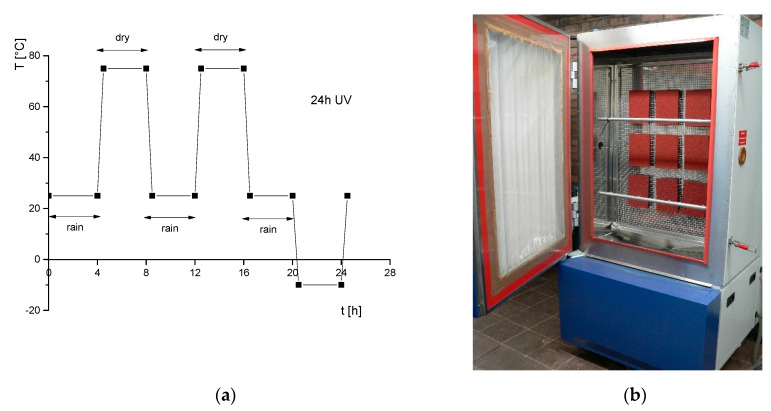
(**a**) 24-h weathering cycle with continuous irradiation [70] and (**b**) weathering chamber prepared for exposing samples of synthetic athletic tracks.

An example of an exposure scheme that includes cyclic thermal mechanical stress is demonstrated in (Figure 3). It has been applied to different materials [36,66,67,68,69]. This 24-h weathering cycle was adapted from a German standard for traffic signs [70]. Long wetting and drying periods of four hours each guarantees deep penetration of rainwater as well as sufficient drying, resulting in the transport of water-soluble extracts out of the polymeric system. At the same time, mechanical stresses are generated on the surface, which can lead to surface crack formation and thus increased diffusion. Similar effects can be achieved with strong temperature cycles. Therefore, high temperature levels are alternated with frost periods. During the whole 24 h, continuous UV irradiance is applied, using UVA-340 nm fluorescent lamps, in accordance with ISO 4892-3 type 1A.

### 4.1. Metals and PAH from Synthetic Sports Surfaces

Artificial turf and other synthetic surfaces have widely been established as replacements for natural grass or cindered pitches and tracks on sports facilities. This is mainly due to substantial improvements in sports performance over the years of development in engineering of such surfaces and less requirements to maintain the desired conditions to enable a more intense usage. Polyethylene, polypropylene, polyamide, polyurethane (PUR), Nylon, styrene butadiene rubber (granules from discarded tires), ethylene propylene diene monomer rubber are frequently used materials for the production of the synthetic components of artificial sports surfaces. Numerous additives (e.g., ZnO as vulcanization agent, PAH from carbon black as UV stabilizer and phenols as antioxidants) are used to tailor the desired properties for this specific application. Due to degradation of the materials throughout its life cycle, there are, however, concerns about posing a risk to the environment by transferring hazardous substances from outdoor sports facilities to percolating rainwater and soil.

**Figure 4:**
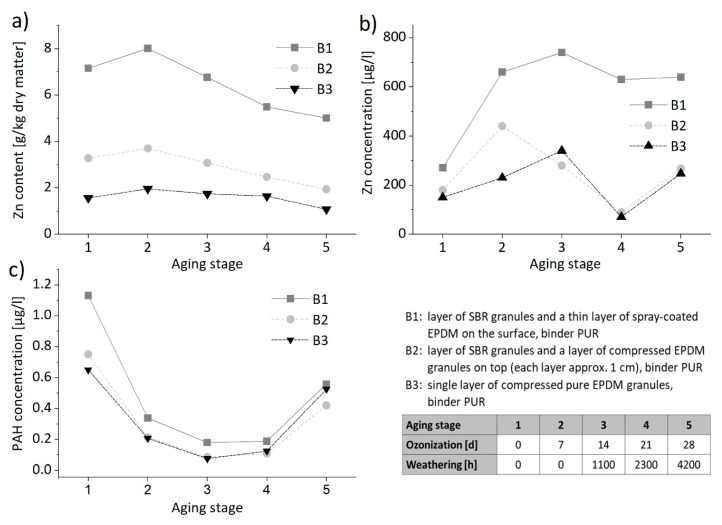
Trend of (**a**) Zn content in solid matter in the course of the artificial weathering of three types of sports floorings (B1 to B3), as well as release of (**b**) Zn and (**c**) PAH measured in the eluates of intermittent batch leaching tests at L/S (liquid-to-solid ratio) of 2 L/kg during artificial weathering.

The leaching behavior with water of individual polymeric components of artificial turf systems and sports surfaces was investigated using batch tests and column tests with and without previous weathering [67,68]. For rubber granules, particularly granules from discarded car tires (i.e., SBR, styrene butadiene rubber), the release of Zn [71,72] and other organic substances [73] was reported. It was found that increasing water exposure decreases the release of inorganic and organic substances, such as Zn and PAH [74]. Due to degradation of the polymeric matrix and the uncovering of leachable components during weathering, Zn release partly increases again [68,75,76]. The topmost layer used for athletic tracks usually consists of SBR and/or EPDM (ethylene propylene diene monomer rubber) granules and PUR as a binding agent. Combining weathering exposure, using a cycle and a weathering chamber as displayed in Figure 3, and leaching tests revealed the long-term contaminant release [68,74] and points to the possible increase of some substances when the polymer matrix begins to degrade (see Figure 4).

With granules coated with PUR to imitate the color of grass, the release of Zn was found to be retarded [77]. In addition to the investigation of the leaching behavior of Zn and organic substances, Li et al. [78] studied the emission of volatile organic substances in the gaseous phase under laboratory conditions as well as natural weathering conditions and found a significant reduction in the latter case.

In contrast to concerns about the environmental compatibility of discarded tire granules, Edil et al. [79] also point to the applicability of this material for the remediation of contaminated water such as landfill leachate, which can be attributed to the high sorption capacity of hazardous volatile organic compounds and was found by performing large scale laboratory tests and field tests.

The increase in leached substances from rubber granules after simulation of mechanical stress using several approaches was reported, e.g., by [80,81].

The complexity of synthetic surfaces on outdoor installations of sports facilities poses a challenge to realism when studying the environmental compatibility, e.g., of polymer-based turf systems or athletic tracks. The mutual influence among the individual components and therefore the appropriate test setup to simulate actual conditions in the field must be taken into account. A concept has been developed to investigate the leaching behavior of such complete synthetic sports surface installations using column tests [67]. All components were assembled in glass columns in accordance with the known or expected field conditions and percolated using demineralized water up to a certain liquid-to-solid ratio. The contaminant release measured in the resulting eluate fractions gives a more realistic picture than do investigations of the individual components. A set of typical outdoor sports surface installations was studied using this tailored test procedure. The contaminant transfer to groundwater was modeled and assessed in terms of the potential environmental impact of selected regulated contaminants [73,82]. Nevertheless, for production control purposes, simpler test methods and separate limit values are needed for the sake of practicability; these are currently being developed. The release of further substances of environmental concern from polymeric sports surfaces and upcoming alternative materials remains to be investigated [83].

### 4.2. Antioxidants from PE

Polymer materials such as polyethylene contain a wide variety of organic additives to improve their stability and their typical properties regarding manufacturing processes and the interaction with environmentally relevant impact factors. Additives used for these applications and requirements include stabilizers, such as antioxidants and other organic compounds. Here, investigations of the residual content of substances within a weathered polymer such as polyethylene for use in water pipelines were performed to evaluate the stabilizer’s migration behavior, where the remaining antioxidant concentration was measured by means of HPLC-DAD and GC-MS, after various aging exposure scenarios [84,85].

### 4.3. Flame Retardants from Thermoplastics

Observations of the effects on the environment of critical substances released by weathering, such as fire retardants, were expressed as a blooming out at the surface, checked by FTIR spectroscopy, and as a loss of surface-related flame-retarding functionality [66].

### 4.4. Carbon Nanotubes from Polymer Nanocomposites

The release of carbon nanotubes (CNT) due to various aging exposures was quantified by radiolabeling the CNTs [86,87]. It was found that irradiation increases the tendency of the studied polymer nanocomposites to release CNTs more than do other environmental impacts, such as shaking in water or rapid temperature changes. The reason for this is suggested to be the photochemical degradation of the polymeric matrix, uncovering CNT.

### 4.5. Additives from Polymer Recyclates

**Figure 5:**
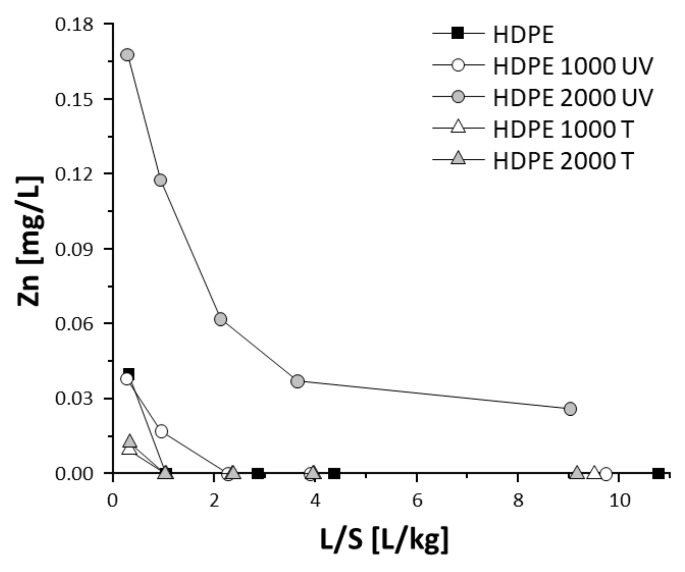
Release of Zn as a function of the liquid-to-solid ratio from high-density polyethylene (HDPE) granules in a column leaching experiment. The greatest release was observed in HDPE particles after 2000 h combined thermal and UV irradiation exposure. Converted to cumulative release, the displayed concentrations correspond to approx. 1% of the total Zn content [88].

Polyvinyl chloride, polystyrene, and polyethylene samples from a plastics recycling plant were exposed to 1000 and 2000 h of artificial aging by photo-oxidation (continuous irradiation without wetting periods) and thermo-oxidation (80 °C) to simulate the fate of potential pollutants in the environment. In general, the investigated plastic particles were smaller than 5 mm and showed increased leaching of organic and inorganic substances after a combined thermal and UV irradiation exposure, see Figure 5 [88]. The release of substances was measured with column percolation tests with water as leachant. Thermal exposure at 80 °C alone was much less effective in accelerating the aging of the polymers, as shown by the release from the samples.

## 5. Comparison of Natural and Artificial Exposure Tests

Natural environmental exposure tests show a broad local and seasonal or annual variability. In contrast, artificial environmental exposure tests enable reproducible results. To simulate the release of critical substances from a material, one can adjust the parameters, aiming at potentially critical environmental exposure conditions, to accelerate or maximize the mass transfer. Thus, to evaluate the ecological menace of a material, one would focus on such a worst-case scenario.

Compared to long-lasting natural environmental exposures, there is a tendency in short-term test approaches to accelerate the exposure in question into a time frame as short as possible. For example, water contact is intensified in leaching tests. However, intra-material mass transfer will affect the release of a chemical into the water. The time frame over which this occurs depends on water uptake and the thickness of the material. With reactive processes, such as biological, chemical, or photochemical processes, it is more difficult to condense the phenomena in question into shorter time frames. Photochemical exposures can be made continuous rather than diurnal, but it would be difficult to increase irradiance without also influencing reactivity, and the time scale of interest is not shortened by very much. Similarly, rates and extents of chemical reactions depend on the concentrations of the reactants, so that increasing the concentration of the reactant could affect the nature of the reaction. Microbial processes, in particular, especially if they involve microbial communities, are the most difficult to artificially accelerate without altering the outcome. Along with microbial processes, increasing the temperature at which a test is conducted can accelerate the phenomena in question without significantly influencing the outcome qualitatively.

Because the mass-transfer processes described above have such an important role in the ultimate release of chemicals into the environment, the effect of exposure cycles on these mass-transfer processes is important to understand and, therefore, might be necessary to reproduce in material-testing protocols. This can include wet/dry cycles, sunlight, freeze/thaw cycles, and ambient atmospheric chemistry that can vary diurnally over scales of days to weeks and seasonally in the same location. For example, leaching tests that involve long periods of contact between the leaching liquid and the material will decrease interfacial mass transfer as the substance in question accumulates in the liquid phase, in turn decreasing intra-material diffusion. In this case, cycling between short wetting periods and longer dry periods might actually increase the release of substances, thus inherently accelerating the process of primary concern while introducing more realism into the testing procedure. In general, any protocol that maximizes intra-material diffusion and/or interfacial mass transfer will accelerate the release of a substance of concern into the relevant environmental compartment. Conversely, any protocol that allows the substance of concern to accumulate in the environmental compartment near the interface with the material will slow its release from the material.

### 5.1. Real Weathering Versus the Weathering Chamber and Soil Bed Test in the Example of the Release of Brominated Flame Retardants

An accelerated aging concept was developed to investigate the release of brominated flame retardants (BFR), which are known for their persistent bio-accumulative and toxic properties, from polymer products to the water and soil compartments [89]. Polystyrene (PS) containing hexabromocyclododecane (HBCD) and polypropylene (PP) containing bromodiphenyl ether BDE-209 were used. These additives are known as substances of very high concern [5,90,91].

The studied PS and PP samples (pieces sized 10 cm × 10 cm × 1 cm) were exposed to a defined weathering schedule in a climate chamber in accordance with a quality requirement of RAL (German National Committee for Delivery and Quality Assurance) [70] (as in Figure 3). BFR were analyzed in the rainwater collected in the climate chamber. An extraction with hexane was performed and the obtained aliquots of the extracts were concentrated and analyzed by GC-MS (BDE 209) or LC-MS/MS (HBCD). Additionally, the total bromine contents were monitored for the aged and untreated samples using laser ablation inductively coupled plasma mass spectrometry (LA-ICP-MS) and X-ray fluorescence analysis (XRF) as a non-destructive and rapid method.

**Figure 6:**
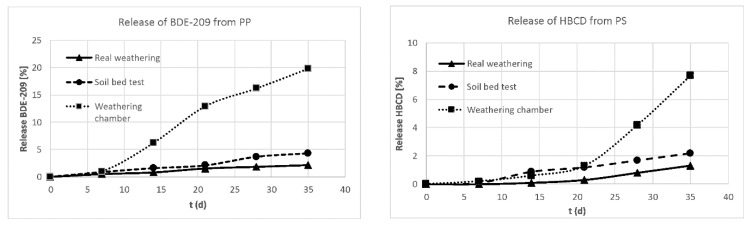
Release of selected polybrominated flame retardants from PP (**left**) and PS (**right**) samples under different environmentally relevant conditions.

Soil bed tests were conducted in an irrigated concrete basin operated indoors at a defined temperature and humidity using a reference soil with high microbial activity. The amount of rainwater was adjusted to promote the growth of a wide range of aerobic microorganisms and varied between 4% and 9% of the soil’s dry mass. The water content was additionally monitored by the weight of the vessel holding the specimens capturing water from raining periods. Correct humidity is a fundamental parameter for biological activity. Five test specimens of each polymer were inserted to a depth of up to half of their length in the soil in the vessel. The microbial activity of the soil was monitored by a reference polyurethane bar, whose haze and tensile strength were analyzed. The release of HBCD and BDE-209 was captured by passive samplers (silicon bars) placed in a distinct distance to the test pieces. After toluene was used to extract the corresponding soil samples and the passive samplers, the extracts were analyzed by GC-MS (BDE 209) or LC-MS/MS (HBCD) [36]. Due to the poor water solubility of the selected polybrominated flame retardants only trace amounts were found by extracting the soil and water samples and the passive samplers. The amount of bromine in the test pieces (aged and stored references) was analyzed by LA-ICP-MS and XRF. Here, an up to 20% decrease of BDE-209 in polypropylene and of HBCD in polystyrene was observed (see Figure 6).

**Figure 7:**
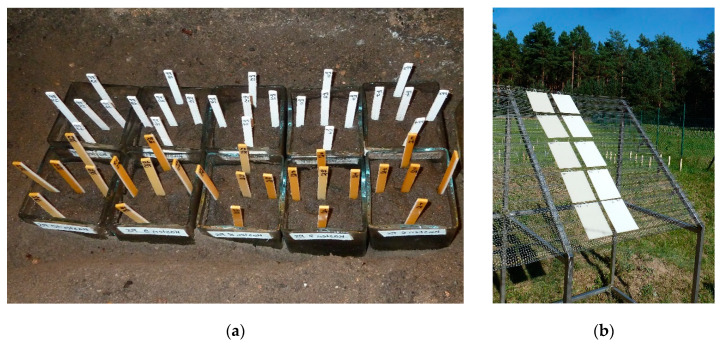
Application of different weathering scenarios: (**a**) soil bed test with flame-protected PP and PS samples and (**b**) real weathering experiments to evaluate the release of polybrominated flame retardants.

For comparison, real time weathering experiments were performed by placing the samples on a weathering rack starting in July 2017 (in a location southeast of Berlin); the rack was aligned in a 45° angle to the south ASTM D1435-13 [92]. The weathering data were regularly recorded by the German Weather Service (DWD). An accelerated release was evaluated by comparing real weathering to climate chamber weathering and soil bed experiments as displayed in Figure 7 [36].

### 5.2. Release of Biocides from Coatings

Coating materials can contain biocides (film preservatives) that are intended to protect the coating film against biodeterioration caused by fungi and algae. These biocides usually act in a water film that is formed on the coatings surface by precipitation or condensed water. Once on the surface, the biocides can be washed off by runoff water.

Reports of the mass transfer of biocides from façade coatings into urban surface waters [93] spurred detailed investigation of biocide leaching from paints and renders. Studies of coatings were performed under laboratory conditions in either simple immersion tests [94,95] or weathering chambers [93,96], in field experiments [39,97,98,99,100], and in settlement areas [93,101,102]. Most of the studies were performed on coatings containing polymeric organic binders, but some studies also included mineral products [103].

The European standard EN 16105 [41] was developed to determine the release of substances from coatings in intermittent contact with water under laboratory conditions [104]. The transport of biocides within the wet coatings is influenced by diffusion [96,104,105], desorption from the coating material [106], and partition between polymeric binder and water [107].

It can be assumed that desorption and diffusion processes follow the same physicochemical principles under laboratory and field conditions. However, experiments under natural weathering conditions include additional, highly variable influencing factors that affect leaching. Therefore, the progression of emission curves from field studies is less consistent and less repeatable than the progression of emission curves from laboratory experiments [39,99,100,108]. UV radiation can cause the photodegradation of biocides [39,99]. In addition, actual exposure of vertical facades to driving rain, but also to sunlight and microclimate, must be considered when results from field experiments are applied to real buildings in an urban environment [109].

The evaluation of risks that can be caused by release of biocides into the environment is a fundamental request of the European Biocidal Products Regulation [110]. For environmental risk assessments, reliable estimation of the expected release of biocides under service conditions is required. This presupposes concepts to use information on mass transfer that is gathered under laboratory or field test conditions to predict chemical release under service conditions. “Transfer functions” are required, which not only need to consider varying exposure to water, but also additional processes, e.g., degradation.

**Figure 8:**
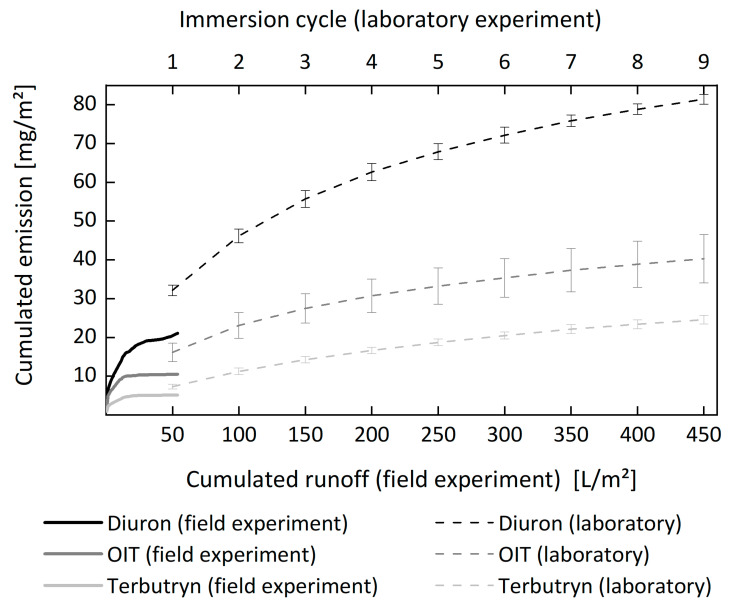
Cumulative emission curves for the biocides diuron, OIT (2-octyl-2H-isothiazol-3-one), and terbutryn from a vinyl acetate-based paint on wood. The amount of contact water is used to compare results from laboratory (EN 16105) and field experiments. During the laboratory experiment, each immersion cycle that consists of two immersion periods of 60 min represents 50 L/m^2^ contact water (upper *x*-axis). Emissions during the field experiment are related to the collected runoff. The laboratory data represent mean values from four experiments, and the error bars indicate standard deviation. The laboratory data represent mean values from four experiments, and the error bars indicate standard deviation.

Emissions of biocides from paints were compared for laboratory tests and field experiments [100,108]. Emissions of the investigated biocides from the paints were much greater in the laboratory experiment performed in accordance with EN 16105 [41] than emissions observed during about two years of outdoor exposure in Berlin (see Figure 8).

**Figure 9:**
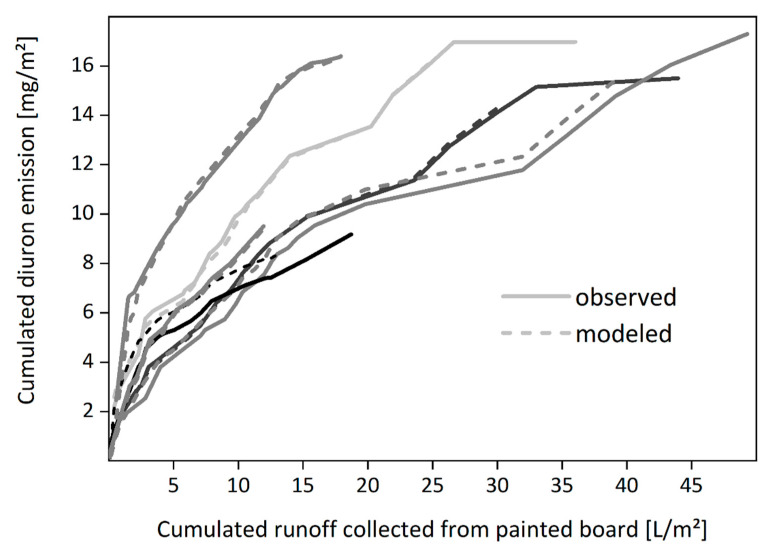
Comparison of experimental emission curves for the biocide diuron and emission curves obtained from a regression model that considers the various actual weather conditions. Boards painted with a vinyl acetate-based paint were exposed to natural weathering at two different sites in Berlin and 60 km north of Berlin and during different periods—resulting in six independent experiments. Runoff was collected after all rain events and analyzed for diuron. Area-related diuron emissions were calculated and demonstrated as cumulative emission curves.

Emission curves from six independent replications of a field experiment demonstrate the influence of variable exposure conditions. However, it was possible to reproduce the different curves using a preliminary regression model that includes the different actual weather data, i.e., exposure to wind-driven rain, temperature, relative humidity, and global radiation (see Figure 9) [108]. Although this can help us understand the effects of weather on leaching processes, it remains a challenging task to apply information on the material properties determined in laboratory tests to service conditions and to include this knowledge in appropriate models and emission scenarios [96,111].

### 5.3. The Time Scale Conundrum

Despite efforts to accelerate the release of a chemical from a material during material testing, there will always be a difference between time scales relevant to actual environmental exposures and the time scale necessary for testing. This might be less important if the test captures the release of most of the chemical over the shorter time scale; in this case, it provides a reasonable estimate of impact, particularly acute impact. If intra-material diffusion governs the rate at which a substance of concern is released; however, the time scale of a test procedure might be too short to estimate environmental impact, depending on the thickness of the material.

Short-term tests can provide useful information even if only a small fraction of a substance of interest is released over the duration of the test.

### 5.4. Long-Term Validation

It is not common for testing methods to be subject to validation procedures to verify the efficacy of the test relative to actual, longer-term environmental exposures. Leaching tests for soil and waste materials, for example, have been compared with results from field lysimeters operated over longer periods [112,113,114], and studies have been conducted to collect rainwater in contact with building surfaces after a longer period in service than would be captured in a standard leaching test [97]. In general, it is worth considering standardizing the field testing of materials after a period in service as part of a quality-improvement strategy to refine test methods. However, such time-consuming and costly studies shall be designed to allow general conclusions, rather than to investigate individual products. It is also important to consider that no straightforward validation is possible, as exposure scenarios are highly dependent on location. Even in a single location, two outdoor exposures at different times may vary greatly. Despite the lack of a general conclusion, the investigation of long-term behavior is important—also from a regulatory point of view—to evaluate if release is falling below a critical level after which no further significant release is to be expected. This decrease of release can be caused either by depletion of the material or by a change in availability.

## 6. Conclusions

Several laboratory tests have been developed in recent decades to investigate the effects of environmental exposure on the release of possible hazardous substances from polymer-based products. As they can focus only on a single or a limited number of test parameters, artificial environmental exposure tests can show high acceleration factors, but always risk decreasing relevance to reality. Furthermore, acceleration factors are sometimes reached under unrealistic conditions, which may allow different chemical reactions or degradation mechanisms than those observed under natural conditions. In contrast to this, natural environmental tests are close to reality per se, but are very specific regarding the exposure site and time. Especially because they are hardly reproducible even in the same location, much more effort should be made to document all possibly necessary exposure parameters to facilitate the interpretation of results.

**Figure 10:**
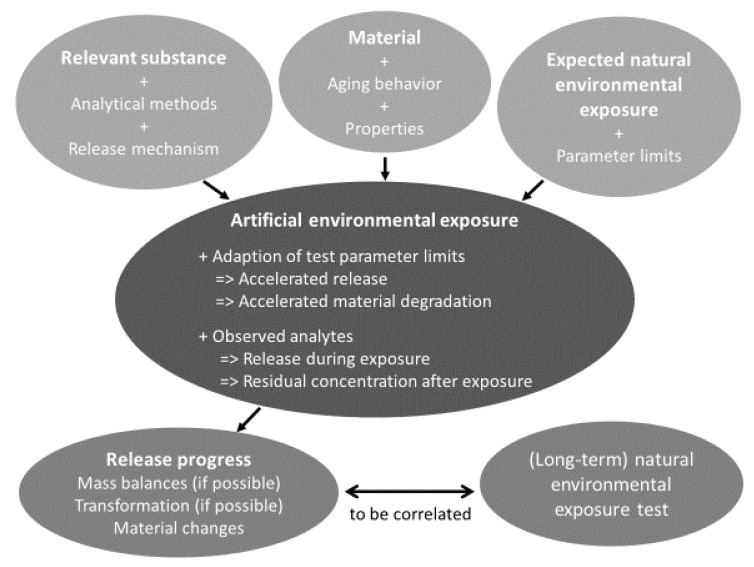
Flow chart for the design of artificial environmental exposure tests. Top: required previous knowledge; center: options of exposure tests; bottom: expected results.

Both approaches are necessary to assess the release of hazardous substances and are best combined to obtain complementary information, see Figure 10. Almost more important than the laboratory test itself is reliable planning before starting, and some basic knowledge about the following is necessary: starting points are the considered substances, the polymer itself, and the expected natural environmental exposure. The release mechanism is substance-specific, and knowledge about it facilitates the choice of a proper analytical method suitable for the examined matrix and sufficiently sensitive and selective. Depending on the polymeric material, one or more degradation mechanism can be relevant for the aging behavior. This is strongly connected to usage scenarios and the parameter limits of the resulting natural environmental exposure.

The artificial environmental exposure tests should be defined based on this knowledge. The test parameter limits should be adapted (within reasonable limits), aiming at accelerated material degradation and maximum release. If the test setup makes it possible to measure both the release during the exposure and the residual concentration within the material after various exposures, mass balances can be established. With useful test durations, the progress of the release, which need not be monotonic, can be followed.

Such release progress from artificial environmental exposure tests must be correlated with (long-term) natural environmental tests to get an impression of time scales in natural environment.
